# Music-based biofeedback to reduce tibial shock in over-ground running: a proof-of-concept study

**DOI:** 10.1038/s41598-021-83538-w

**Published:** 2021-02-18

**Authors:** Pieter Van den Berghe, Valerio Lorenzoni, Rud Derie, Joren Six, Joeri Gerlo, Marc Leman, Dirk De Clercq

**Affiliations:** 1 Biomechanics and Motor Control of Human Movement, Department of Movement and Sports Sciences, Ghent University, 9000 Ghent, Belgium; 2 Department of Arts, Music and Theatre Sciences, IPEM, Ghent University, 9000 Ghent, Belgium

**Keywords:** Preventive medicine, Risk factors

## Abstract

Methods to reduce impact in distance runners have been proposed based on real-time auditory feedback of tibial acceleration. These methods were developed using treadmill running. In this study, we extend these methods to a more natural environment with a proof-of-concept. We selected ten runners with high tibial shock. They used a music-based biofeedback system with headphones in a running session on an athletic track. The feedback consisted of music superimposed with noise coupled to tibial shock. The music was automatically synchronized to the running cadence. The level of noise could be reduced by reducing the momentary level of tibial shock, thereby providing a more pleasant listening experience. The running speed was controlled between the condition without biofeedback and the condition of biofeedback. The results show that tibial shock decreased by 27% or 2.96 g without guided instructions on gait modification in the biofeedback condition. The reduction in tibial shock did not result in a clear increase in the running cadence. The results indicate that a wearable biofeedback system aids in shock reduction during over-ground running. This paves the way to evaluate and retrain runners in over-ground running programs that target running with less impact through instantaneous auditory feedback on tibial shock.

## Introduction

### Real-time feedback on tibial acceleration during treadmill running

Gait retraining intends to alter a motor pattern that has become habituated over many years^1^. Gait retraining has been put forward as a method to reduce or treat injuries in distance runners^2,3^. Various studies have focused on the reduction in tibial shock (i.e., the axial peak tibial acceleration)^1,4–7^, presumably because the magnitude of the tibial shock has been associated with tibial stress fracture susceptibility. Evidence for this association is provided in female distance runners^8^. Other case–control studies failed to observe a clear difference in groups of runners with and without a history of tibial stress injury^9,10^. Nevertheless, gait retraining on a treadmill with the intention of lowering the impact loading has led to fewer running-related injuries (62% lower injury risk) in novice runners^2^. These runners could reduce the maximum instantaneous vertical loading rate of the ground reaction force^2^, an impact measure that has been correlated with tibial shock during level over-ground running^11,12^. In several studies^4–7^, a reduction in tibial shock has been stimulated by providing biofeedback while participants were running on a treadmill (Supplementary information file, supplement 1). For instance, Crowell and colleagues provided biofeedback that comprised a visual stream of the axial component of tibial acceleration in real-time^5^. The biofeedback was shown to the runners using a screen in front of a treadmill during a single session of gait retraining in the laboratory. The concept of real-time biofeedback for lower impact running was further developed by Wood and Kipp, who provided auditory biofeedback in the form of pitched “beeps” scaled relative to a runner’s baseline of peak tibial acceleration^13^. This simple auditory feedback was found to be equally effective for tibial shock reduction compared to the visual biofeedback during a short run on a treadmill in the laboratory^14^. Another lab-based study used a combination of visual (traffic lights) and auditory (pitched beeps) feedback modalities in runners screened for high tibial shock^7^. The authors reported a reduction in tibial shock of 3.28 g or 31% after completing a multi-sessions program of gait retraining on a treadmill^7^. All these lab-studies demonstrate the effectiveness of biofeedback at reducing tibial shock. Importantly, they pave the way to study lower impact running through such biofeedback in real-world running environments.

### From pitched beeps to music with superimposed noise

Efforts have been made to apply music-based biofeedback^15,16^. Music can act as a strong motivator for walking and running^17–19^, so music may be implemented to achieve a pleasant or motivational stimulus. Music-based biofeedback has been explored in people with brain damage after ischemic stroke or traumatic brain injury, to stimulate weight-shift training in patients with impairment in balance function^20^. Music-based biofeedback has also been proven effective to steer posture parameters while performing a weightlifting task^16^. There are indications that music can be used as stimulus in a context of reinforcement learning^16^. The application of music to reduce tibial shock fits well in the context of distance running as about half of the recreational runners regularly train with music^18^. In short, the development and testing of a wearable music-based biofeedback system will advance the ecological validity of studying runners who engage in gait retraining.

Based on the above considerations, we have developed a wearable music-based biofeedback system. It consists of a measurement module and a feedback module. The measurement module detects tibial shock and cadence in real-time using accelerometers^12^. The feedback module generates shock-dependent pink noise which is superimposed onto synchronized music to stimulate lower impact running^12,21^. The feedback subsystem has the capability to synchronize the tempo of the music with the running cadence in real-time, which has been experienced as motivating^22^. The use of synchronized audio in an exercise program consisting of locomotor activities has improved adherence to physical activity^23^, emphasizing the idea that interaction with music is empowering^24,25^. The beats per minute of the music continuously adapt to the steps per minute of the runner^21^, so the music-based biofeedback system allows for cadence-induced changes if desired by the user. A high momentary level of tibial shock results in a high level of noise. If the runner adopts a self-selected gait adaptation which reduces tibial shock, then the noise level is reduced and the acoustical quality of the music improves. In terms of reinforcement learning this creates a punishment/reward dynamic. The whole wearable music-based biofeedback system opens the possibility to test whether runners can reduce the cyclic shock experienced in the lower extremities with the aid of a runner-friendly form of auditory biofeedback.

### A self-discovery approach for lower impact running

In previous studies, explicit instructions about running technique have been given to participants with the intention of reducing impact^26,27^. In these studies^27–33^ groups of shod runners were asked to substantially increase their running cadence (i.e., steps per minute) or to change to an anterior foot strike pattern^6–28^^.^. Besides explicitly imposing a particular change in running technique, a more personalized approach is to let the runner discover his or her own motor strategy of lower impact running with the use of biofeedback as in^7,13^. In one such preliminary report, Morgan and colleagues observed a systematic increase in running cadence when groups of about ten runners received visual or auditory real-time biofeedback with the intention of reducing the magnitude of an unspecified component of peak tibial acceleration on a treadmill^14^.

### Aim and hypotheses

We sought (1) to determine the potential of music-based feedback to induce lower impact running in a group of runners who had high tibial shock and (2) to investigate if an eventual shock reduction would be achieved by a clear increase in the running cadence. Runners with high tibial shock experienced shock magnitudes in the highest one-third of the population. A systematic review indicated that feedback on tibial shock has been effective in reducing tibial shock while running on a treadmill^34^. Consequently, our first hypothesis was that runners with high tibial shock would be able to decrease their level of tibial shock while running over-ground with the use of continuous, real-time, auditory biofeedback on tibial shock at a stable running pace. In a first step toward understanding how runners adapt to real-time auditory biofeedback outside the traditional laboratory, the running cadence was included in the analysis. Therefore, our second hypothesis was that the group of high impact runners would spontaneously increase the running cadence in an attempt to reduce tibial shock at a stable running pace.

## Methods

### Participants

For screening purposes, a total of 88 runners were recruited from the Flemish running population. Analogous to Clansey and Crowell and colleagues^4,7^, the current study targeted runners experiencing high tibial shock. In this case, the runners with a one-legged averaged value of tibial shock in the highest one-third of the 88 screened runners were contacted to take part in the intervention. The first ten runners who volunteered were selected. An a priori power analysis (GPower; α = 0.05, an effect size of 1.5, paired testing) estimated a required sample size of at least seven (n = 7). The effect size in the power analysis was based on results of a treadmill-based gait retraining program for runners experiencing high tibial shock^4^. Based on the email response time, the first ten participants who agreed to participate were selected and engaged in the intervention. The sample size is in line with previous studies that offered a single session of real-time feedback on tibial shock to a group of runners^6,13,35^ (Supplementary information file, supplement 1). The ten selected participants were at least 6 months injury-free and ran in non-minimalist footwear^36^. They ran at least 15 km/week distributed over at least two sessions at the time of the study. Training habits were questioned (Table 1). All participants signed an informed consent approved by the ethical committee of the Ghent University hospital (Bimetra Number 2015/0864). The methods were carried out following their guidelines and regulations. Informed consent was obtained from the participants to publish the information/image(s) in an online open-access publication. This consent was also obtained from test leaders who might be recognizable in some images.

**Table 1:** Participants’ characteristics: anthropometrics and self-reported training habits.

Variable	Mean	SD	Range	
			Minimum	Maximum
Body height (m)	1.70	0.07	1.59	1.79
Body mass (kg)	67.7	7.4	56.2	82.1
Age (year)	33	9	24	49
Training volume (km/week)	29	12	15	50
Training speed (m∙s^−1^)	2.88	0.31	2.36	3.33

### Research design

**Figure 1:**
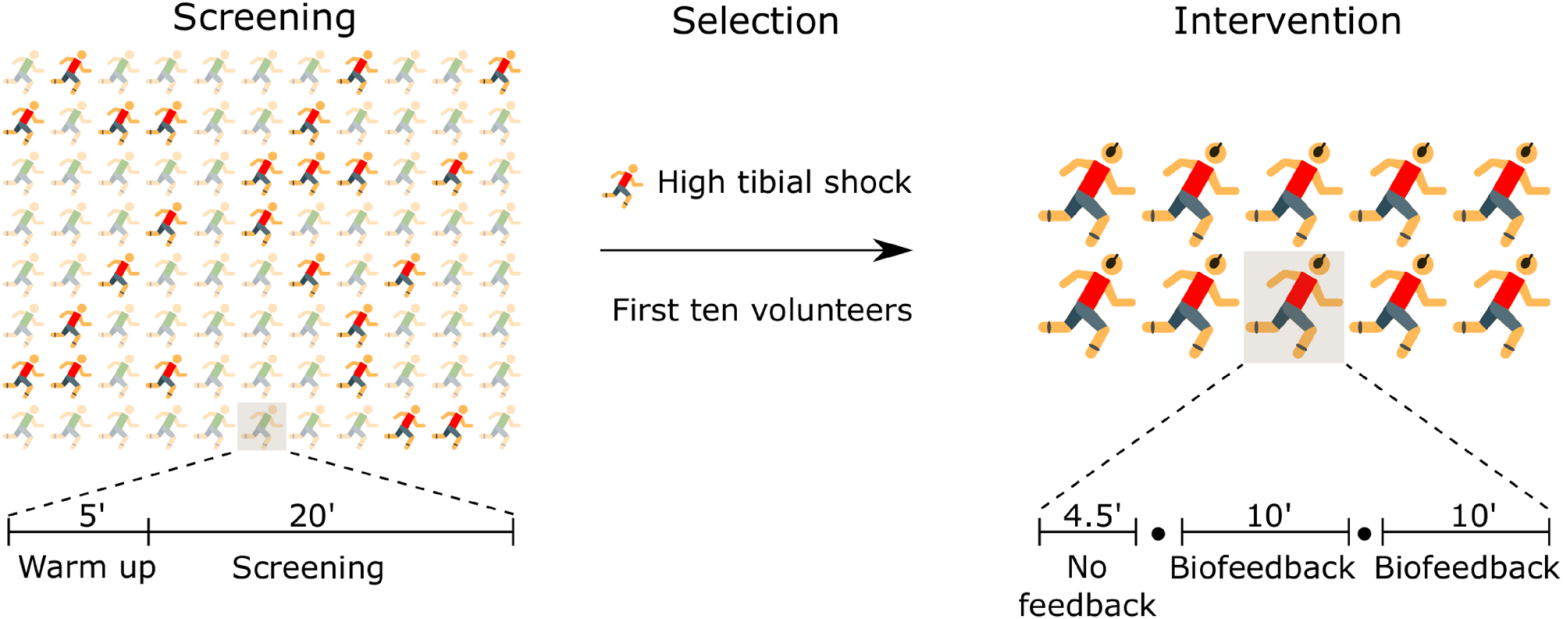
Schematic overview of the experimental design involving two running sessions (screening and intervention). A red icon represents a distance runner with high tibial shock. A filled circle indicates a system check and self-selected rest. Tibial shocks were detected in both sessions. The music-based feedback module was activated in the biofeedback condition.

The quasi-experimental study was unblinded and used a pre-post design without controls (Fig. 1). Two over-ground running sessions were completed in the runner’s regular sportswear at a speed of 3.2 ± 0.2 m∙s^−1^, a common speed range to evaluate endurance running^2,6,12,27,38^, while instrumented with a wearable system that was developed for real-time identification of tibial shock and auditory biofeedback on tibial shock. First, we identified the runners with high tibial shock following a screening session (October 2017—February 2018) in a sports laboratory. Then, a supervised intervention session with auditory biofeedback on tibial shock took place at an indoor track-and-field site (January—March 2018, supplementary video 1). This study employed a within-subjects design to examine changes in tibial shock. The days of individual testing were supplemented (digital supplementary file, datasheet). The time required to complete the two sessions was about 2 h (2 · 1 h). The days between the sessions ranged from 59 to 138 (89 ± 28, mean ± SD).

### Screening session

#### Set-up

A test leader instrumented the standing participant with a stand-alone backpack system. A 7″ tablet (Windows 10) was fixated to a stripped backpack and connected via USB port to a microcontroller (Teensy 3.2, PJRC). The microcontroller was connected to two lightweight, tri-axial accelerometers (LIS331, Sparkfun, Colorado, USA;1000 Hz; ± 24 g) to measure tibial acceleration bilaterally^12^. The test leader who instrumented the participant was part of a research team with varying experience and expertise level; from a last-year student in sports sciences to a post-doctoral researcher. The tibial skin was pre-stretched by strappal tape at ~ 8 cm above the left and right medial malleolus to minimize unwanted oscillations of the skin in the vertical direction during impact^7,12^. Thereafter, an accelerometer fitted in a shrink socket with a total mass less than 3 grams was firmly attached to the anteromedial aspect of each lower leg by means of non-elastic zinc oxide tape (Supplementary information file, supplement 2 a). The axial axis of the accelerometer was aligned visually with the longitudinal axis of the lower leg before mounting. The tape was tightly fastened by one of the test leaders to the limit of subject tolerance. The applied alignment has been common practice for research involving tibial acceleration in running^7,12,37^.

#### Procedure

An initial warm-up and familiarization period of five minutes was given along an oval track (circa 32-m length ∙ 5-m width). Participants subsequently ran for circa 20 min. The running speed was monitored on a trial-by-trial basis by timing gates spanning 6-m near the middle of a straight section. The first five satisfactory trials of each foot were collected for processing. Trials were discarded if the running speed fell outside the set boundary of 3.2 ± 0.2 m∙s^−1^.

#### Data processing

The recorded tibial accelerations were imported for signal processing via custom-built MATLAB scripts. Tibial shock magnitudes corresponding to the first five contacts on a force platform were averaged for each foot side and per participant. Unfiltered magnitudes of tibial shock were preferred because the tibial shocks detected by the biofeedback system were derived from the raw signal for the instantaneous auditory biofeedback. The leg with the highest value was retained. We evaluated the distribution of tibial shock in the group of screened runners and invited the runners who experienced shock magnitudes in the highest one-third of that population.

### Intervention session

#### Set-up

The single-session intervention was supervised and took place at a track and field facility (supplementary video 1). The accelerometers of the wearable system were re-applied to the participant’s lower leg (supplementary information file, supplement 2 b). The manner of attachment of the accelerometer in the intervention session was intended to be identical to that of the screening session. The simple mounting technique has resulted in repeatable mean values of the tibial shock between running sessions^12^. The participant wore an on-ear headphone (HD25-ii, Sennheiser, Wedemark, Germany).

#### A smart music player for real-time music-based feedback on tibial shock

A peak detection algorithm repetitively detected the magnitude and timing of tibial shock in each leg^12^. A custom-built JAVA program operated on the backpack system and detected a peak every time the axial acceleration exceeded 3 g with no higher axial acceleration value measured in the next 375 ms. This simple algorithm was based on a peak detection algorithm taken from a previous gait retraining study^7^. The magnitudes and timings were transmitted in real-time through Open Sound Control to a MAX/MSP patch that was built with the intention of providing music-based biofeedback in real-time^21^. Real-time in this context means with negligible delay. For instance, when a new magnitude of tibial shock was detected, the auditory manipulations were executed in the same stride cycle.

**Figure 2:**
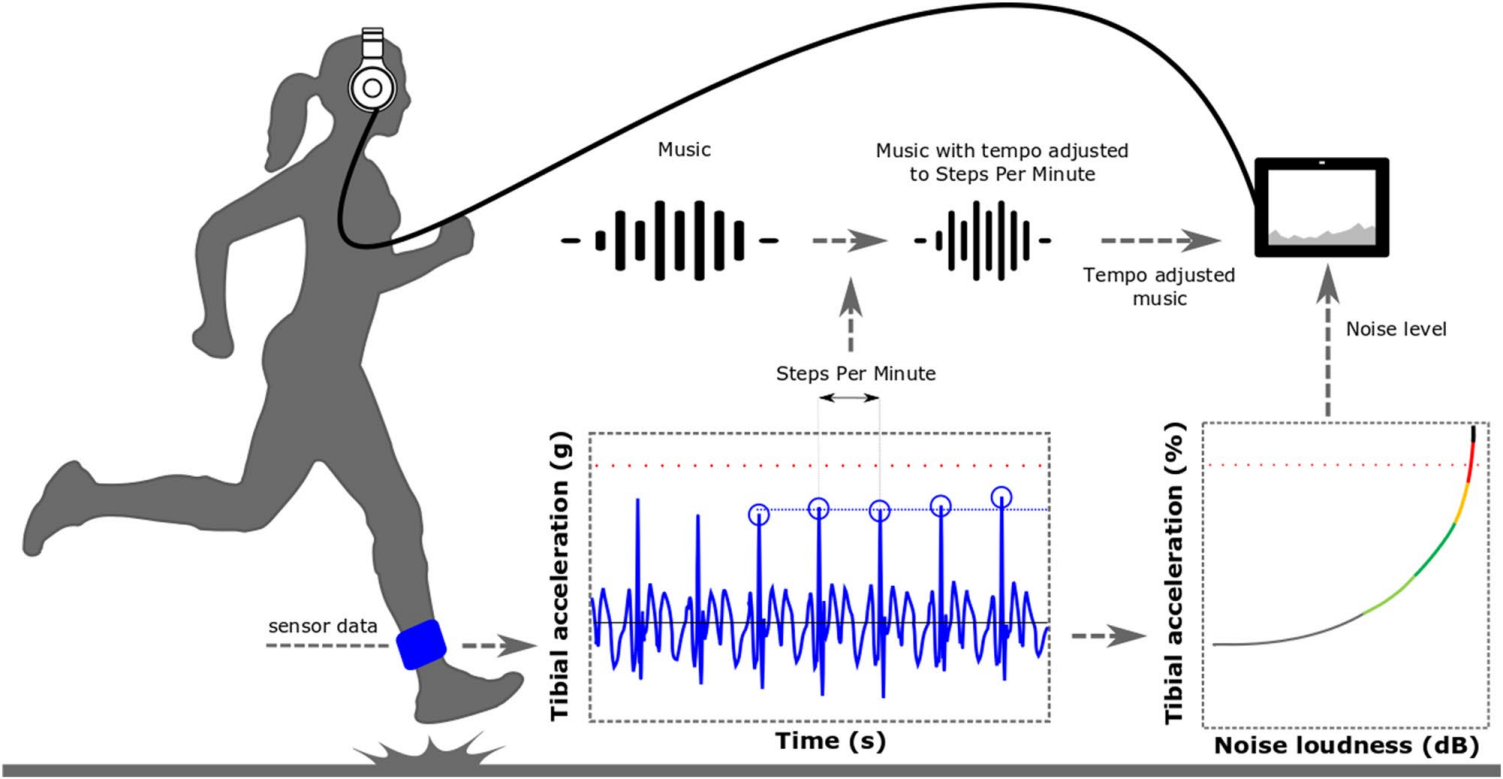
Schematic representation of the biofeedback system’s main components for continuous biofeedback on tibial shock (axial peak tibial acceleration). An interaction loop of the smart music player that provided the auditory biofeedback in real-time and that continuously accounted for (in)voluntary alterations in the running cadence by aligning the tempo (beats per minute) of the music to the cadence (steps per minute) of the runner. The red horizontal line indicates the baseline tibial shock. The five most recent values of tibial shock are averaged and mapped to a discretized level of noise loudness, which is added to the music playing.

The real-time, continuous, auditory biofeedback consisted of commercially available music tracks with superimposed pink noise of variable loudness (Fig. 2). The loudness of the noise depended on the momentary level of tibial shock of the leg that experienced the greatest mean shock in the baseline measurement. The five last values of that leg’s tibial shock were averaged through a 5-point moving average to account for inherent step-to-step variability in tibial shock^7^. That momentary level of tibial shock was mapped using an empirically validated fitting to obtain a distinct level of noise loudness^21^. Six discrete loudness levels (0, 20, 40, 60, 80, 100% of noise) were created for good discretization (supplementary audio, fragment 1)^21^, thereby accounting for inter-subject differences in the decoding accuracies^39^. The loudness levels were calculated as a percentage of the root-mean-square amplitude level. So, the upper limit of 100% corresponded to noise with the same amplitude as the root-mean-square amplitude level of the music. Shock values below the target resulted in music only, meaning without pink noise (0% of noise). The target of minus ~ 50% of the baseline tibial shock was taken from previous gait retraining studies^4,5,7,14^.

The running cadence was derived from the timings of the tibial shocks detected during running. We intended to repetitively align the tempo of the music (i.e., the beats per minute) to the running cadence (i.e., the steps per minute) in the biofeedback condition^22,40^. This real-time synchronization prevents the runner from adjusting his or her cadence to the tempo of the music and is based on the idea that interaction with music is empowering^24,25^. Music of a preferred genre (pop, rock, electronic dance, swing, world) was chosen by the participant. A music database consisting of seventy-seven tracks with a clear beat in the tempo range of running at sub-maximal speed was created (supplementary information file, supplement 4). Songs with the right tempo were selected by a smart music player that instantaneously and continuously adjusted for a change in the running cadence. Music tempi were manipulated up to ± 4% of the steps per minute without pitch shift^21^ (supplementary audio, fragment 2). When a change in steps per minute exceeded this tempo shift for eight seconds, another song started playing at a tempo that more closely resembled the altered running cadence. An illustrative audio fragment of a change in a music track was supplemented (supplementary audio, fragment 3). The momentary ratio of the music-to-motion alignment is described by the ratio of the running cadence (steps per minute) to the tempo of the music (beats per minute). The ratio should be close to 1 when the beats per minute of the music are aligned with the steps per minute of the runner.

#### Procedure

Once bilaterally instrumented with the accelerometers and the backpack (Supplementary information file, supplement 2 b, c), participants ran an initial 4.5 min at ~ 3.2 ± 0.2 m/s. This warm-up period functioned as the no feedback condition wherein no auditory feedback on tibial shock was provided. In the software patch, the baseline tibial shock of the leg exhibiting the highest overall tibial shock was automatically determined for a sequence of 90 s (≈ 1 lap of 289 m) in the middle of the no feedback condition. Before the biofeedback condition started, the runners (i) were familiarized with the different levels of noise loudness by listening to the discrete noise levels going from minimum to maximum and vice versa (supplementary audio, fragment 1); (ii) chose their preferred sound volume; (iii) chose their preferred music genre; (iv) received verbal instructions in mother tongue: “This may be very difficult, but I would like you to try your best to concentrate on the task throughout the entire intervention. Listen carefully to the distorted music. Try to run with the music as clear as possible without any distortion at all. If impossible, keep the music distortion as low as possible by modifying your running technique. The amount of distortion is linked to your tibial shock. The music stops playing when the trial is over.” So each runner was instructed to find a way to run with a lower level of tibial shock. However, to elicit self-discovery strategies, no instructions were given on how to reduce the shock magnitude^7,13,14^. An illustrative fragment of auditory biofeedback with the different noise levels was supplemented (supplementary audio, fragment 4).

Biofeedback was provided for 20 min in total with a pause after 10 min. The instructions were repeated during the pause of self-selected duration. The software was configured in such a way that the music and the detection of tibial shock automatically stopped after the set period of time. The runner finished the lap and met the test leader at the checkpoint (Supplementary information file, supplement 2 d). Subsequently, the accelerometers and the backpack were removed from the lower limb. Meanwhile, the runner reported if he or she perceived any difference in the amount of superimposed noise in the biofeedback condition (yes/no). If so, we asked to describe the perceived change in running technique. An estimation of exercise intensity was obtained by asking the runner to give a score (from 1 to 10; from very easy to maximal effort) based on the session rating of perceived exertion scale^41^. The subject’s score was collected ~ 5 min after the end of the running session. Three participants did not report their level of exertion. Accelerometer data were continuously acquired during the no feedback and biofeedback conditions. Lap times were hand clocked throughout the session to derive the running speed of a lap. Verbal feedback about the running speed was given on a lap-by-lap basis to the runner.

#### Data processing

The proportion of the pink noise generated during the 20-min biofeedback run and the detected tibial shocks were imported for processing using custom-built MATLAB scripts. The tibial shock values of each individual were extracted for a period of 90 s in both the no feedback and biofeedback conditions. The period of the no feedback condition corresponded to the period of the baseline measurement. The tibial shocks belonging to the biofeedback condition were extracted for another period of 90 s at the end of the biofeedback run. Post hoc inspection of all the registered peaks revealed that the peak detection algorithm worked sub-optimally by occasionally detecting false-positive peaks. The values belonging to the falsely identified peaks were post hoc excluded (supplementary information file, supplement 3). The time period at the end of the biofeedback run was chosen for comparison, like that seen in previous research^5,6,13,14^. We wanted to obtain a representative level of overall tibial shock per participant compared to previous research on gait retraining (i.e., 5 to 20 footfalls) (supplementary information file, supplement 1). Therefore, the values of the tibial shock (g) and the running cadence (steps per minute) of the detected footfalls that belong to the no feedback and the biofeedback conditions were retained for the larger 90 s time period. The analyzed peaks were considered to be indicative of foot–ground contact. The number of analyzed footfalls was respectively 125 ± 10 and 132 ± 7, mean ± SD. Hence, it becomes possible to show the distribution in tibial shock, for example, in someone maximally responding to the music-based biofeedback. The time between sequential tibial shocks was used to derive the steps per minute in order to assess the running cadence. The average running speeds of the no feedback and biofeedback conditions were calculated for each participant using the lap times clocked at the indoor track. The running speed was also determined for those laps corresponding to the extracted tibial shocks.

For further statistical analysis, the tibial shock, the running cadence and the running speeds were averaged per participant for each condition. Wilcoxon exact signed-rank tests were used for comparison due to the low number of participants. Tibial shock, running cadence and running speeds were compared between the no feedback condition and the biofeedback condition. Tibial shock and running cadence were tested one-tailed (p_1_) because of the directional hypothesis. The Pearson correlation coefficient was calculated post hoc between the session rating of perceived exertion as reported by the runner and the difference in tibial shock. The alpha level was set at 0.05 (SPSS). The effect size r_ES_ was calculated by dividing the absolute z-score by the square root of the total number of observations, being r_ES_ =|z|/√20. Guidelines for r_ES_ are that a small effect is 0.1, a medium effect is 0.3, and a large effect is 0.5^42^. The individual metrics can be retrieved online (digital supplementary file, datasheet). The reported values are mean ± SD.

## Results

### Tibial shock in the intervention session

**Figure 3:**
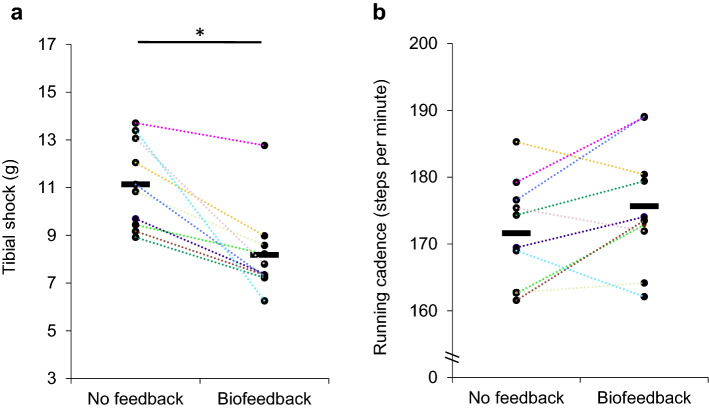
(**a**) The axial peak tibial acceleration representing the tibial shock and the (**b**) running cadence for the [left] no feedback and [right] biofeedback conditions. Every color is a different participant. The short horizontal line indicates the mean level of the variable of interest in a condition. * indicates *p* < 0.05.

Tibial shock was 11.14 ± 1.83 g in the no feedback condition. The individual averages of tibial shock ranged from 8.92 g to 13.71 g between the participants. Tibial shock scores were reduced by 27% to 8.19 ± 1.79 g (p_1_ = 0.001, z = −2.803, r_ES_ = 0.627 (large), mean negative rank = 5.50, absolute range: −0.94 to −7.14 g; relative range: −7 to –53%) in the biofeedback condition (Fig. 3**a**), and this without guided instruction on gait modification.

**Figure 4:**
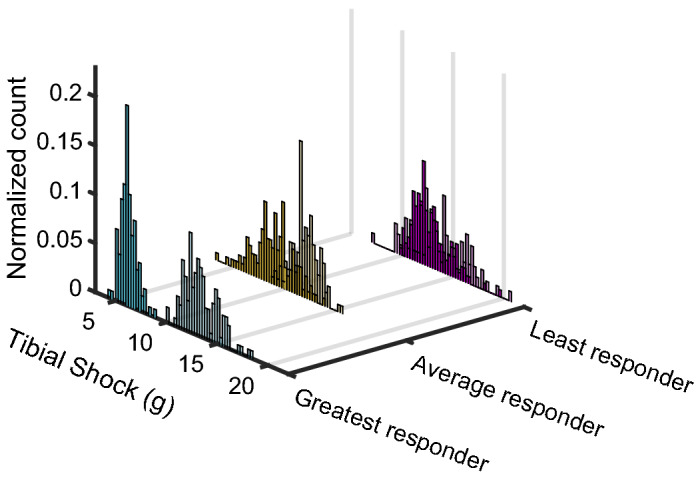
Histogram of the tibial shock magnitudes in the analysis period (90-s) for the no feedback (dark) and biofeedback (light) conditions in the greatest, average and least responders. The footfalls of each runner in a condition have been normalized to the number of total footfalls in that condition.

**Figure 5:**
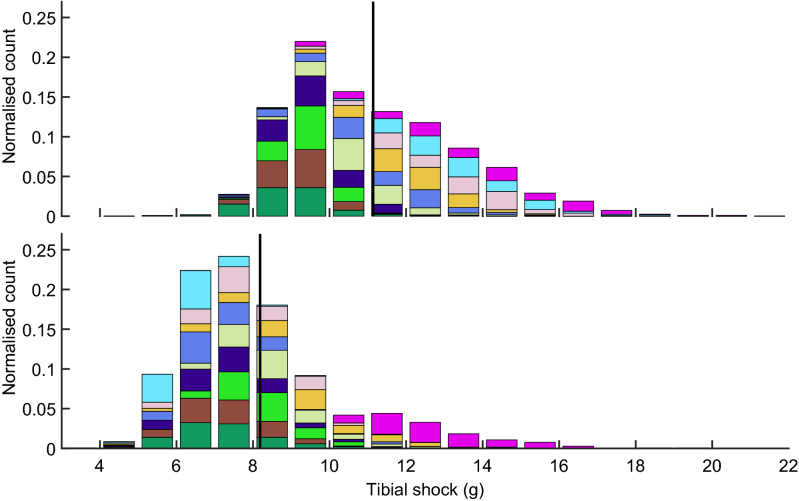
Histogram of the tibial shock magnitudes in the analysis period for the [upper panel] no feedback condition and the [lower panel] biofeedback condition. Each color represents a participant (n = 10). The number of footfalls within a single bin has been normalized to the total number of detected footfalls in that condition. The solid vertical line indicates the tibial shock averaged for all footfalls in that condition.

Figure 4 shows the distribution in tibial shock for the average, most and least pronounced responder. While there is an overall decrease in the magnitude of tibial shock in these three runners, few footfalls had a tibial shock that would still be categorized as high. Figure 5 shows the group’s distribution in tibial shock for both conditions.

### Music-based biofeedback characteristics

**Figure 6:**
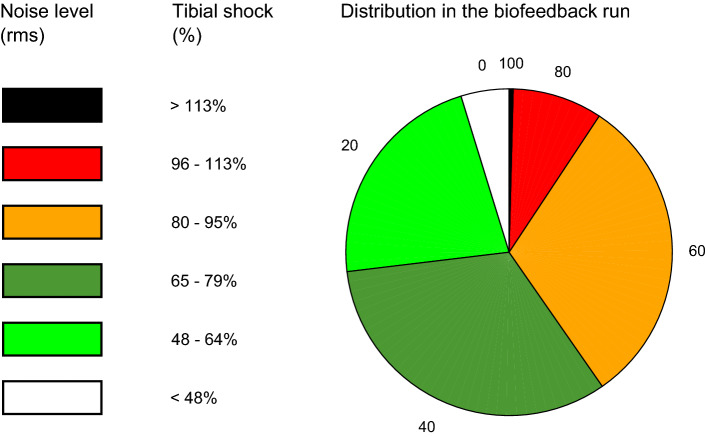
The proportion of the pink noise generated during the 20-min biofeedback run for the group of high impact runners. Level 0 represents the ‘music only’ category without superimposed noise. The level of noise loudness added to the synchronized music has been subdivided into 5 categories. Each level of noise loudness corresponds to a level of tibial shock relative to the baseline *g*-value of the runner, which was determined during the no feedback condition. The value corresponding to 100% of tibial shock is identical to the value of tibial shock determined in the no feedback condition. Tibial shock is here synonymous to the axial peak tibial acceleration, rms: root mean square.

The average momentary ratio of the running cadence to the music tempo was 1.01 ± 0.01 in the 20-min period of biofeedback and 1.02 ± 0.04 in the time period selected for comparison. The noise loudness to the synchronized music varied from zero to maximum on the group level (Fig. 6). This means that tibial shocks did occur both below the target (0% of noise) and above the baseline level of tibial shock (100% of noise). All noise levels were experienced in this group of runners with high tibial shock (Fig. 6). The individual proportions of the noise levels have been supplemented (Supplementary information file, supplement 5). The questioned runners responded quasi-immediately after completing the running session to have perceived a change in noise loudness or quality of the audio during the biofeedback run (digital supplementary file, datasheet).

### Temporospatial characteristics

Figure 3b shows the individual evolution in the running cadence between the conditions of no feedback and biofeedback. The increase of 4 steps per minute or 2.3% in the running cadence was not statistically significant (p_1_ = 0.065, z = − 1.580, r_ES_ = 0.353 (moderate), positive mean rank =  + 6.14). The running speed in the 4.5-min no feedback and 20-min biofeedback runs were respectively 3.15 ± 0.12 m∙s^−1^ and 3.13 ± 0.15 m∙s^−1^, and did not differ significantly (*p* = 0.52, z = − 0.71, r_ES_ = 0.159 (small)). The respective running speeds for the laps chosen for tibial shock comparison were 3.18 ± 0.15 m∙s^−1^ and 3.04 ± 0.10 m∙s^−1^, and did not differ statistically (*p* = 0.090, z = − 1.72, r_ES_ = 0.385 (moderate)). In addition, the running speeds remained within the a priori permitted boundary of ± 0.20 m∙s^−1^.

### Perceived exercise intensity

The mean and median scores of the session rating of perceived exertion were respectively 4 (somewhat hard) and 3 (moderate) with individual values ranging from 2 to 9 (digital supplementary file, datasheet). In this cohort, the participant reporting the highest rating of perceived exertion also reported the lowest combined training volume and training speed. The perceived exertion did not correlate to the absolute (*p* = 0.460, r = 0.337) nor relative (*p* = 0.561, r = 0.268) decreases in tibial shock, suggesting that the attained level of exertion did not influence the achieved reduction in tibial shock.

## Discussion

The purpose of this proof-of-concept study was twofold: (1) to determine if real-time, continuous, music-based feedback on tibial shock helps to reduce the shock magnitude during over-ground running at an instructed and common running speed, and (2) to examine if runners with high tibial shock systematically increase the running cadence in response to the real-time feedback. A single-session intervention was performed at an instructed running speed with pre and post measurements in a screened group of runners. The runners who participated in the intervention session had an averaged value in one of the limbs of at least 9.7 g in tibial shock when screened in the laboratory. A wearable system provided real-time auditory feedback on a modifiable mechanical parameter to stimulate lower impact running in a controlled, indoor training environment.

### Key implications and discussion regarding the reduction in tibial shock

In support of our first hypothesis, runners with high tibial shock decreased their tibial shock by − 27% or − 2.96 g while running over-ground with the music-based biofeedback. This is the first study performed over-ground in which high impact runners realized shock reduction with the use of unimodal biofeedback. Our findings build on previous research about gait retraining in high impact runners^4,7,29^, and support the limited literature documenting that self-discovery strategies to achieve shock reduction are effective^7,13^. For instance, Clansey and colleagues carried out a randomized controlled trial and reported a decrease of 3.28 g in male runners with high tibial shock who completed multiple sessions of continuous real-time feedback on tibial shock at the controlled running speed 3.7 m∙s^−1^^7^. The decrease in tibial shock we found corresponds to the decrease reported by Clansey and colleagues in the experimental group, though the present study was performed at the slightly lower running speed of ~ 3.2 m∙s^−1^ and in a single-session design. The mixed-sex runners in the present study could run a total of 25 min at 3.2 ± 0.2 m∙s^−1^ and all achieved shock reduction at the end of the biofeedback run. These runners’ shock reduction did not correlate to the reported session rating of perceived exertion. Hence, a substantial reduction in tibial shock is achievable in a heterogeneous group of recreational runners with the aid of a wearable biofeedback system. The participants were informed about the aim of the intervention (i.e., shock reduction) and they were aware of the fact that an auditory element was linked with the tibial shock. However, no explicit instructions about gait modification were given.

### Key implications and discussion regarding the expected increase in running cadence

The spontaneous self-adaptation in response to the music-based feedback permitted the runners to find their own solution to cover ground with less tibial shock magnitudes, without reducing the running speed. Self-induced changes in running cadence were possible because the music’s tempo was continuously and successfully synchronized to the runner’s cadence. Contrary to our second hypothesis, a reduction in tibial shock was not accompanied by a systematic increase in the running cadence (or a decrease in step length because the running speed remained stable). A preliminary and treadmill-based study has reported a systematic reduction in an unspecified component of peak tibial acceleration when providing real-time auditory feedback in response to that peak tibial acceleration, that was accompanied by a systematic increase in running cadence of 2 steps per minute or 1.4%^14^. In the present study the cadence response between the participants was more variable (Figs. 3, 4). The discrepancy in a systematic change in step frequency between study results highlights the fact that more work is needed to fully understand the motor strategy or strategies for tibial shock reduction. For instance, another way to reduce tibial shock may be a change in the discrete foot strike pattern.

An anterior change in foot strike pattern has been found in rearfoot runners with high tibial shock who completed a treadmill-based, multi-week, retraining program by means of visual and auditory biofeedback on tibial shock^7^. In the current study performed in an over-ground running environment and at a slower running speed, half of our participants claimed to have tried a non-rearfoot strike in the biofeedback condition. Only a single runner declared to have maintained a forefoot strike until the end of the run. Almost all of the participants (9 out of 10) claimed to have performed a rearfoot strike near the end of the biofeedback condition. Based on our observations and on the comments made by the participants, we speculate that the real-time feedback on tibial shock elicits gait alterations with inter-individual differences in kinematic adaptations. Consequently, the gait alterations may influence shock attenuation strategies. A shift from active shock attenuation to more passive mechanisms has, for instance, been proposed as possible adaptation during prolonged running at a submaximal intensity^43^. When providing biofeedback on the axial peak tibial acceleration, the shock attenuation may rely more heavily on the active mechanisms (e.g., eccentric muscle contractions, changes to joint angles, and modulating limb stiffness) than passive deformation of the body tissues. Future research may verify our speculations because 3D kinematics, head nor sacral acceleration were measured.

### Discussion regarding the targeted reduction in tibial shock using a music-based approach

We attribute the large effect size obtained in our study to the use of reinforcement. Previous studies that used a manipulation of music to modulate gait parameters have relied on a steering paradigm that is based on reinforcement learning^25,44^ according to which people tend to modify their behavior in order to maximize reward and recursively minimize error (i.e., distance from the target behavior). In this specific case, we sought to reward the runner by providing a way of obtaining maximum acoustic quality of the synchronized music. The rewarding effect of running with only music, thus without superposition of pink noise, occurs if the target is reached. Surprisingly, a 50% reduction in tibial shock was reached only for 4.8% of the 20-min biofeedback run. The quote “*I heard several noise levels, but I never heard music without noise*” of a participant illustrates this finding. Even the greatest responder could not fully supress the level of superimposed noise (i.e., so that only synchronized music would be heard) for the majority of the time (supplementary information file, supplement 5).

Many studies on gait retraining with biofeedback aimed to reduce the runner’s baseline value in tibial shock with 50%^4–7,14,45^. But this relative threshold was difficult to achieve or to maintain in the present study. According to our data, a more realistic and relative target for the population of interest seems to be approximately − 30% in tibial shock. Given that some gait adaptations felt unnatural when trying to achieve a 50% reduction in tibial shock, a more feasible target of shock reduction may also counteract the slight discomfort reported by several participants at the end of the run. Nevertheless, more retraining sessions are likely required before the self-discovered gait pattern is perceived as natural. A cohort of runners with high tibial shock namely reported that the new gait pattern felt natural by the end of the sixth retraining session, comprising the instruction to run softer and the use of real-time feedback about tibial acceleration^4^.

Next to feasibility, it is debatable whether an extreme target of − 50% in tibial shock is required to be clinically relevant. Chan and colleagues have executed a randomized controlled trial with one-year follow-up and reported fewer running-related injuries in novice runners who completed a gait retraining program on treadmill^2^. Even within the multifactorial nature of injury development, their findings are promising to consider gait retraining as a preventive strategy for running-related injuries in distance runners who appear to be at risk for injury^2^. Their multi-week gait retraining program was performed on an instrumented treadmill with an instruction intended to reduce the vertical impact peak force. The group of runners who engaged in the retraining program could reduce the instantaneous vertical loading rate of the ground reaction force by about 15 to 18%, estimated by manual digitization of the results visualized in Fig. 4 of that publication, and depending on the running speed tested^2^. Such a reduction in vertical loading rate might be linked with a reduction in tibial shock because of the moderate correlation between the vertical loading rate and the tibial shock during over-ground level running^11,12^. Multiple lab studies have provided real-time feedback on tibial shock and did report a substantial reduction in tibial shock and in vertical loading rate post-retraining^4,7,45^. A reduction of about 30% in both tibial shock and vertical loading rate has been achieved by runners with high tibial shock post-retraining in a laboratory setting^45^. So, a more feasible target of approximately -30% in tibial shock relative to the baseline measurement may still have potential to reduce or to treat running-related injuries in at-risk runners during level over-ground running. The evidence for an association between measures of impact over time and running-related injuries has been conflicting^9,10,46–51^. Nevertheless, guided usage of a wearable biofeedback system that induces and retains substantial impact-like reduction over time may have clinical implications for injury risk management.

### Limitations

The self-selected or fixed running speed has been held constant in gait retraining studies that aim to reduce tibial shock^4,7,45^. The instructed and lap-by-lap monitored speed of 3.2 ± 0.2 m∙s^−1^ was slightly above the group’s self-reported training pace for their typical distance runs (Table 1). It was still less than the running speed of 3.7 m∙s^−1^ imposed by Clansey and colleagues^7^ in male runners during the 20-min retraining sessions.

The instructed running speed of the present study may affect results since it influences the absolute magnitude of impact measures in the time domain, such as tibial shock and the instantaneous vertical loading rate^12^. Nonetheless, Chan and colleagues showed that the vertical loading rate was lowered at multiple running speeds after gait retraining^2^.

Individualization of the instructed speed to the training speed of the participant’s typical distance run may further increase the ecological value of gait retraining. Given that the session rating of perceived exertion indicates the exercise intensity^41^, we estimate that the running session was generally performed near the first ventilatory threshold. The average score of 4 on the session rating of perceived exertion scale resembles a physical effort that was “somewhat hard” in this group of mixed-sex runners. Even the participant who reported the highest score of 9 was able to reduce tibial shock. No linear relationship was found between shock reduction and perceived exertion. These results suggest sufficient attention is required for lower impact running with the use of the biofeedback at the instructed speed. This may not be the case at higher exercise intensities, for instance, when the runner needs to cope with maintenance of the running pace during exhaustive runs.

The exploration of gait adaptations might affect running economy. Tibial shock reduction has led to more oxygen being consumed whilst running on treadmill in a single session of gait retraining^35^. In contrast, a multi-sessions program comprising real-time feedback on tibial shock resulted in a clear reduction in tibial shock without affecting the running economy^7^. Future research may verify the hypothesis of a temporary decrease in running economy in an over-ground setting because oxygen consumption was not measured in the present study.

The design of this study does not allow confirmation of whether the synchronised music influences the tibial shock via the biofeedback system. The results can only be attributed to the auditory biofeedback, being the combination of synchronized music and superimposed noise. Besides a positive effect of music to training adherence, there might also be other effects because of the ability of music to distract from a task^52^. It could be further investigated which kinds of music perform best in a retraining context.

In line with previous studies^4,7,45^, the study was conducted in healthy runners who demonstrated a characteristic previously associated with a history of tibial stress fracture in distance runners. Therefore, these findings are not necessarily applicable to injured runners nor to runners with relatively low magnitudes of tibial shock. The selected group of runners had high tibial shock relative to a screened cohort. That inclusion criterion may be a reason for the discrepancy in the absolute reduction of tibial shock (*g*) between studies with and without a focus on high impact runners only^4–7,13,14,35,45^.

The changes in outcome cannot be fully attributed to the intervention without comparator group. The lack of a control group raises questions about whether the reduction in tibial shock is the result of the continuous real-time feedback or the awareness of the purpose of the feedback (i.e., shock reduction). Verbal information was given to elicit self-discovery strategies without the provision of direct instructions (e.g., “run softer”, “land with a toe-strike”) that may influence tibial accelerations. Although we find it unlikely that explicitly instructing people to “decrease your tibial shock” without clinician or accelerometry guided feedback would result in a substantial shock reduction at the end of a running session, it remains unknown and unexplored.

### Future directions

The wearable system can instantaneously detect and sonify tibial shock. The next step is to determine the effectiveness of the biofeedback system in an over-ground gait retraining program with a control group. A gait retraining program lasting multiple weeks usually involves fading of the feedback stimulus^2,4,45^. Analogous to the gradual removal of the continuous and visual stream of tibial acceleration during the last four sessions by Crowell and colleagues^4^, the continuous auditory feedback may be faded over time to facilitate internalization and persistence of an altered gait pattern. An assessment of motor retraining was beyond the scope of this study, there it normally requires about six to eight sessions to enhance retention of the alterations in the movement pattern^4,7,30^, but could be incorporated in gait retraining protocols.

A possibility to retrain runners in more natural environments eliminates the need of exclusive retraining in laboratory/clinic settings. As such, runners might easily implement the auditory biofeedback-driven approach of retraining, given some technical improvements (e.g., wireless accelerometer connected to a miniaturized processing device) and adequate speed control. The smart music player might also benefit from a feedback protocol that promotes motor learning in a retraining program consisting of multiple sessions.

## Conclusion

Our experimental study without controls shows that a substantial reduction in tibial shock can be stimulated with the use of continuous music-based biofeedback. If the runners are aware of the direct link between the tibial shock and the clarity of the music, there is no need to impose a particular gait modification with the intent of shock reduction. The proof-of-concept supports the idea that lower impact running is possible in an over-ground environment by providing instantaneous auditory information on biomechanical data via a wearable biofeedback system.

## Supplementary Information.

Supplementary Video 1.

Supplementary Information.

Supplementary Table 1.

Supplementary Audio Fragments.

## Data Availability

The dataset used for statistical analysis and several exemplar audio fragments are available in the supplementary materials.
