# Supplementary material for: Music-based biofeedback to reduce tibial shock in over-ground running: a proof-of-concept study
Source: Sci Rep. 2021 Feb 18;11:4091. doi: 10.1038/s41598-021-83538-w (PMC7892879; doi:10.1038/s41598-021-83538-w)
Supplement: Supplementary file 2 — Supplementary Information. [file 41598_2021_83538_MOESM2_ESM.docx]

# Supplementary information file

Music-based biofeedback to reduce tibial shock in over-ground running: A proof-of-concept study

Pieter Van den Berghe^1,*^, Valerio Lorenzoni^2^, Rud Derie^1^, Joren Six^2^, Joeri Gerlo^1^, Marc Leman^2^, and Dirk De Clercq^1^

Supplement 1. Overview of single session experiments for running retraining by means of augmented feedback on Peak Tibial Acceleration (PTA) that targeted shock reduction within one running session. The studies are sorted alphabetically. B: Baseline measurement; P: Post-measurement; TM: Treadmill.

| **Study**  **(year)** | **Participants: Sample size (♂:♀), physical state, running volume, age** | **Retraining  protocol** |  | **PTA component**  (sampling rate) | **Extracted data** | **Results** | **Setting** | **Control  group** |
| --- | --- | --- | --- | --- | --- | --- | --- | --- |
| Creaby & Smith (2016) | 22:0  Healthy  >10 km/wk  18-45 y | Dosage: 1 session, 10 min. feedback  Feedback: visual or verbal  Instruction: “run softer”, “make your footfalls quieter” |  | Axial  (1500 Hz) | 5 strides in the last 10 s of each min. | B: 5.74 ±2.25 g  P: 5.34 ±1.93 g | TM | No |
| Crowell (2010) | 0:5  Healthy  > 32 km/wk  26 ±2 y | Dosage: 1 session, 2x10 + 5 min feedback  Feedback: visual  Instruction: “run softer”, try to get value below the line on the monitor |  | Axial  (1080 Hz) | First 20 strides from the last 15 s. | B - P: not specified in text  mean effect -32% (range: -60%, +6%) | TM | No |
| Morgan & Kipp (2015) | 10:11  Healthy  > 16 km/wk  18-25 y  Rearfoot strikers | Dosage: 1 session, 5 min feedback  Feedback: Visual or auditory  Instruction: Not specified |  | Not specified  (not specified) | 10 s from the last 30 s. | B: 4.76 ±1.22 g  P: 3.87 ±0.71 g | TM | No |
| Wood & Kipp (2014) | 3:6  Healthy  > 10 km/wk  20 ±1.5 y  Rearfoot strikers | Dosage: 1 session, 2x 5 min feedback  Feedback: Auditory  Instruction: ”Run without beeps”, “keep pitch of beeps as low as possible” |  | Biofeedback: 3D resultant  (612 Hz)  Reported:  2D vector sum | First 20 strides from the last 30 s. | B: 5.9 ±0.7 g  P: 5.3 ±0.8 g | TM | No |
| Townshend et al. (2016) | 12:0  Healthy  >10 km/wk  23.4 ±5.3 y | Dosage: 1 session, 20 min feedback  Feedback: visual or verbal (clinician guided)  Instruction: “run softer” |  | Axial  (1500 Hz) | 5 strides in last 10 s. | mean effect: -1.9 g (95% CI: -10.9 - -54.3%) | TM | No |

Supplement 2. **a** Representative attachment of the lightweight sensor over the distal medial tibia. The tibial skin is pre-stretched by non-elastic tape. **b** Bilateral attachment of the tri-axial lightweight accelerometer in a participant. **c** The wearable backpack system for provision of music-based biofeedback.


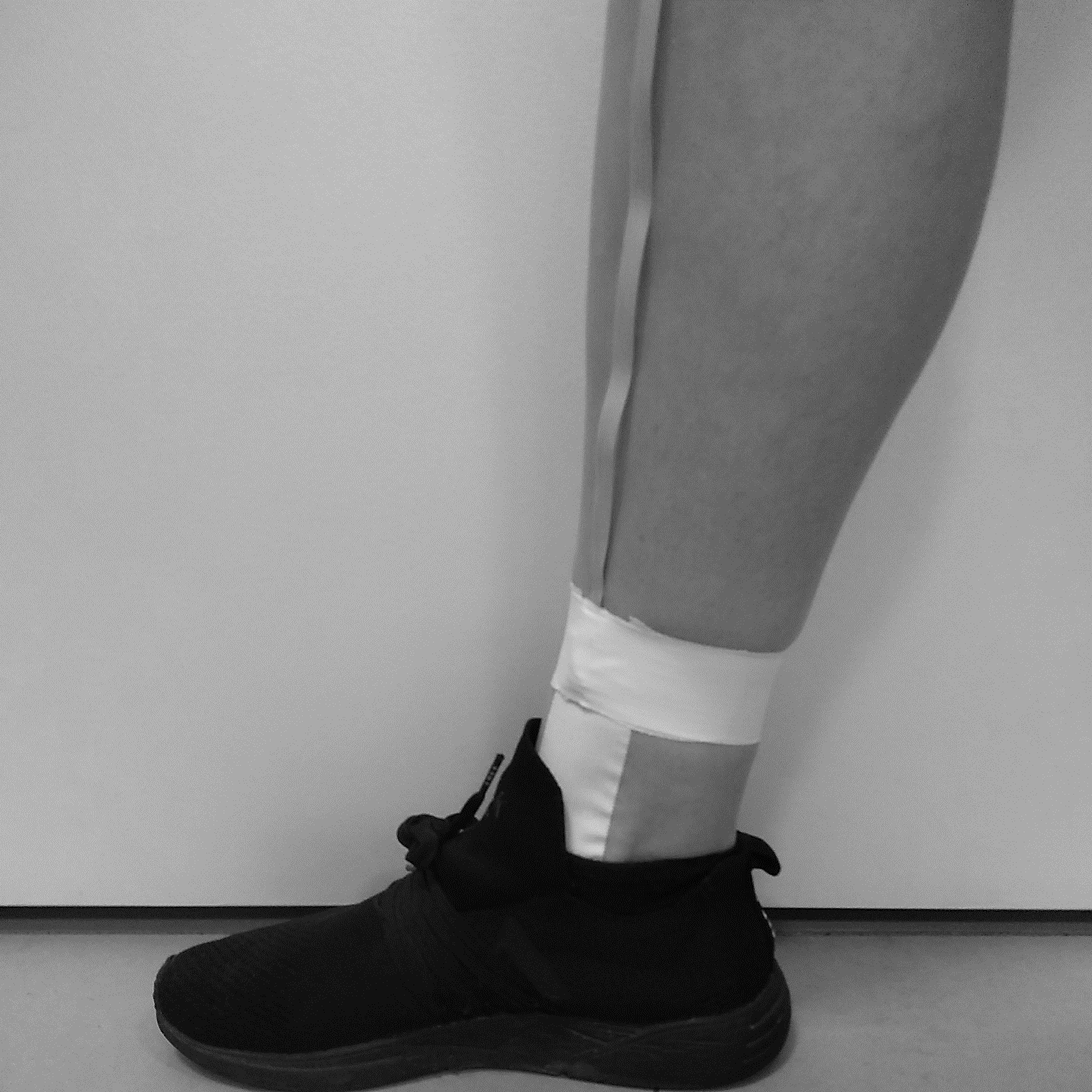


**a**

**c**


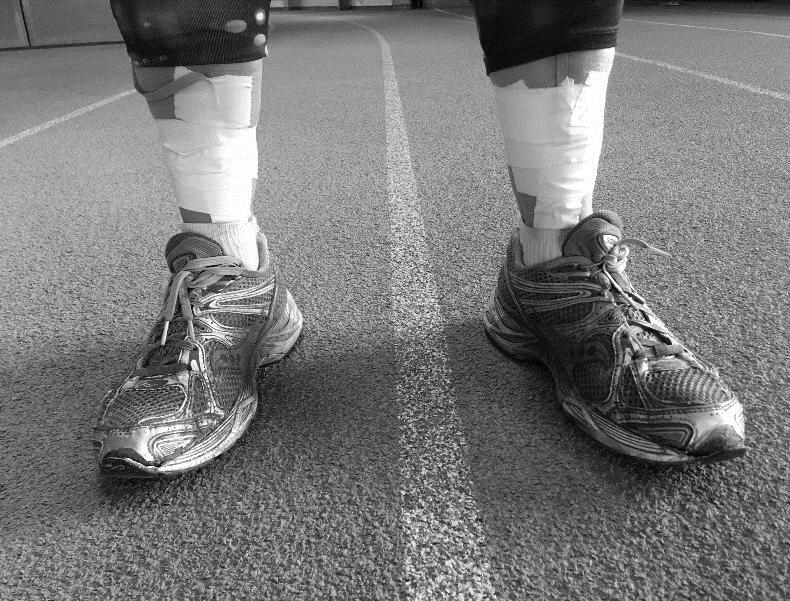


**b**


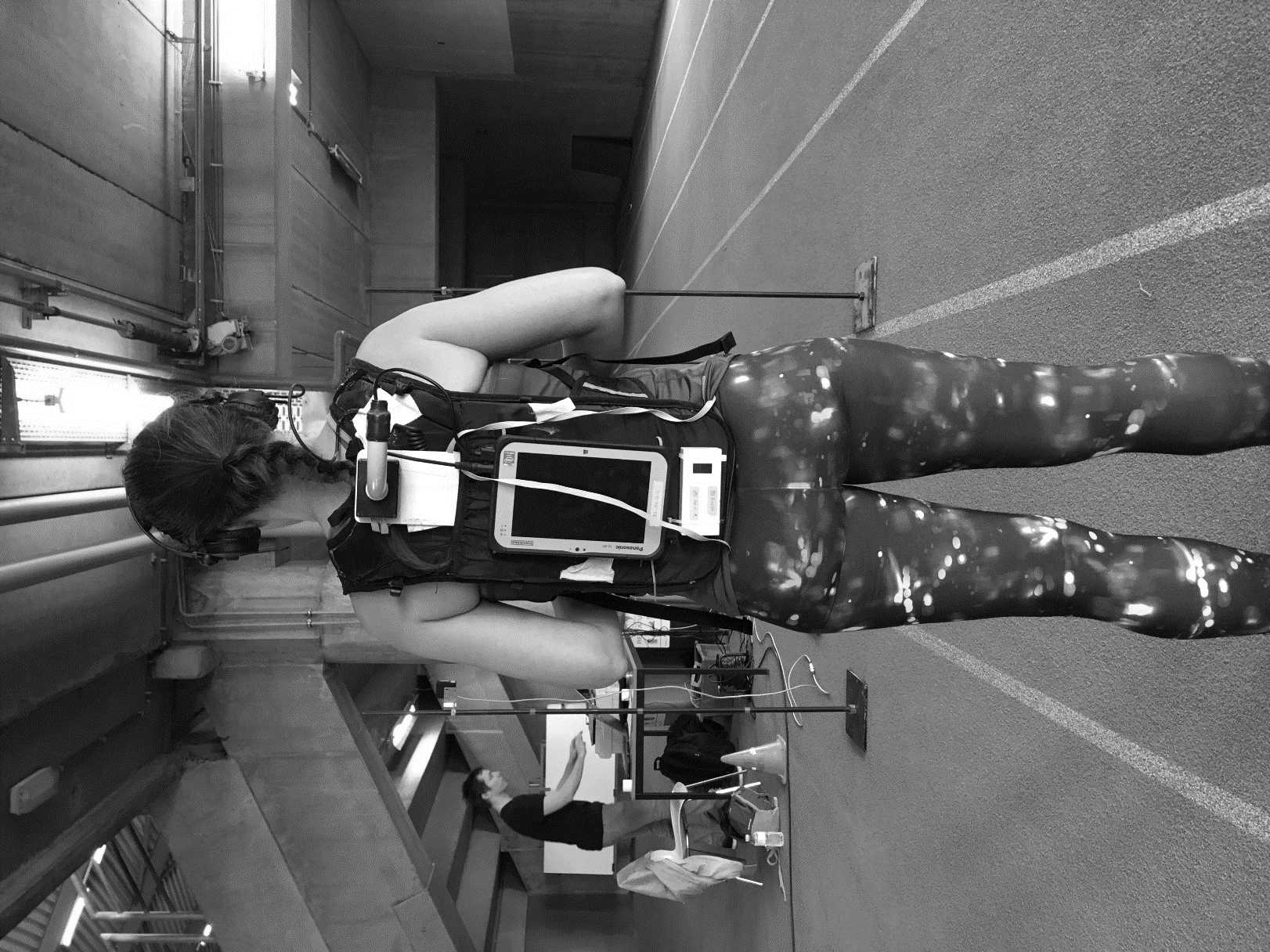


Supplement 3. Exclusion of the falsely identified peaks. The false-positive peaks could be detected for removal using two exclusion criteria based on the step frequency that was calculated using the timing of the detected peaks of the axial tibial acceleration: (1) Step frequency should not differ by more than 20 % from the average step frequency of the trial; (2) Step frequency should not differ by more than 10 steps per minute from the contralateral foot for peak values smaller than 4 g. If a faulty data point was detected, the previous and the next four data points were also removed. Namely, in these instances, the live biofeedback given to the participant would also have been faulty. In the no feedback condition only one participant had to be corrected in this way, leading to a loss of 5% of the collected data during the 90-s time period of averaging for statistical comparison. Two participants were corrected in the biofeedback condition, affecting 1% and 3% of data. The affected data, however, lies out of the 90-s time period for the between-conditions comparison.

Supplement 4. List of musical stimuli. Sorted according to ascending beats per minute (BPM) with the genre arranged in alphabetical order.

| **BPM** | **Artist** | **Title** | **Genre** |
| --- | --- | --- | --- |
| 127.72 | Parov Stelar | Hotel Axos | Dance |
| 130.05 | Benny Bennasi | Satisfaction | Dance |
| 135.98 | Pitbull and Christina Aguilera | Feel this Moment | Dance |
| 140.83 | Daft Punk | Superheroes | Dance |
| 142.00 | Rocksopp | Tricky Tricky | Dance |
| 149.95 | Major Lazer | Roll The Bass | Dance |
| 157.56 | Crystal Fighters | I Love London | Dance |
| 159.98 | Venetian Snares | Chainsay Fellatio | Dance |
| 162.09 | U96 | Love Religion | Dance |
| 167.65 | The Labrats | Give My Soul (Kruder&Dor remix) | Dance |
| 173.88 | Sub Focus | Follow the Light | Dance |
| 175.2 | Rudimental | Waiting All Night (feat. Ella Eyre) | Dance |
| 177.11 | DJ Fresh | Golddust | Dance |
| 180.00 | Madcap | Coming Your Way copy | Dance |
| 185.00 | High Contrast | The Beat Don't Feel The Same | Dance |
| 122.00 | Mika | Relax (Take It Easy) | Pop |
| 135.49 | The Killers | Human | Pop |
| 140.00 | dEUS | The Soft Fall | Pop |
| 140.2 | P!nk | Who Knew | Pop |
| 150.26 | Paolo Nutini | New Shoes | Pop |
| 153.45 | Jermain Jackson Pia Zadora | When The Rain Begins To Fall | Pop |
| 156.97 | Queen | Dont Stop Me Now | Pop |
| 159.9 | Pharrell Williams | Happy | Pop |
| 160.07 | Kaiser Chiefs | Everyday I Love You Less And Less | Pop |
| 162.00 | Wham! | Wake Me Up Before You Go-Go | Pop |
| 164.95 | Radiohead | Jigsaw Falling into Place | Pop |
| 169.54 | The Cure | The Boys dont cry | Pop |
| 174.10 | Kenny Loggins | Footloose | Pop |
| 179.00 | One Direction | Kiss You | Pop |
| 180.19 | KNaan Ft. Adam Levine | Bang Bang | Pop |
| 198.15 | Rihanna | Pon De Replay | Pop |
| 106.02 | Arno | Oh La La La | Rock |
| 122.59 | Status Quo | Whatever You Want | Rock |
| 133.32 | The Kinks | There Will Be No Next Time | Rock |
| 141.50 | XTC | Generals and Majors | Rock |
| 145.90 | The Clash | Clampdown | Rock |
| 150.28 | Arctic Monkeys | Teddy Picker | Rock |
| 153.07 | Franz Ferdinand | Love Illumination | Rock |
| 155.96 | Kaiser Chiefs | Never Miss a Beat | Rock |
| 158.21 | The Strokes | Reptilia | Rock |
| 160.00 | Customs | Justine | Rock |
| 162.00 | Blondie | One Way or Another | Rock |
| 167.56 | The Black Keys | Lonely Boy | Rock |
| 177.97 | The Gaslight Anthem | 45 | Rock |
| 179.54 | Aerosmith | Fever | Rock |
| 186.00 | Ok Go | Don't Ask Me | Rock |
| 206.05 | R.E.M. | It's the End of the World | Rock |
| 101.96 | Carl Douglas | Kung Fu Fighting | Swing |
| 123.16 | Arabesque | In The Heat Of A Disco Night (I Think I Like It) | Swing |
| 131.70 | Grace Jones | I Need a Man | Swing |
| 136.52 | James Brown | Stone Cold Drag | Swing |
| 139.81 | Brainstorm | Lovin Is Really My Game | Swing |
| 143.52 | James Brown | I Got You (I Feel Good) | Swing |
| 149.02 | The Herbaliser | The Missing Suitcase | Swing |
| 156.33 | Ike & Tina Turner | What You Get Is What You See | Swing |
| 157.46 | Bill Haley | See You Alligator | Swing |
| 166.62 | James Brown | Who's Afraid of Virginia Woolf_ | Swing |
| 173.12 | The Dirty Dozen Brass Band | That's How You Got Killed Before | Swing |
| 180.72 | Dick Dales & His Del-Tones | What'd I Say | Swing |
| 185.40 | The Mavericks | As Long As There's Loving Tonight | Swing |
| 191.31 | Adam Ant | Goody Two Shoes | Swing |
| 196.97 | Casey MacGill & The Spirits of Rhythm | Rhythm | Swing |
| 209.13 | Vannebos Dixielandband | Five Foot Two | Swing |
| 088.02 | Manu Chao | Me Gustats Tu | World |
| 124.24 | Bob Marley | Jamming | World |
| 130.31 | Outback | Baka | World |
| 140.03 | Hilight Tribe | Giovani | World |
| 140.17 | Tabla Beat Science | Palmistry | World |
| 144.98 | Hilight Tribe | Kuku | World |
| 155.10 | Oro Solido | Maria Se Fue | World |
| 156.60 | The Bambi Molester | Theme from Slaying Beauty | World |
| 158.45 | Young Dubliners | The Rocky Road to Dublin | World |
| 160.23 | Staff Benda Bilili | Avramandole | World |
| 170.16 | Oro Solido | Abusadora | World |
| 173.60 | Mohammad Al Hasan Abo Abid | Back Streets of Cairo | World |
| 176.0 | La Banda Gorda | Que No Se Acabe el Mambo | World |
| 189.71 | Keb' Mo' | Angelina | World |

Supplement 5. The proportions of noise loudness generated by each of the 10 participants in the biofeedback condition. At the zero noise level, only music was provided. The loudness of pink noise increased exponentially from level 20 to level 100. Each color indicates a level of noise loudness; white: 0, light green: 20, dark green: 40, orange: 60, red: 80, black: 100 % of noise loudness.


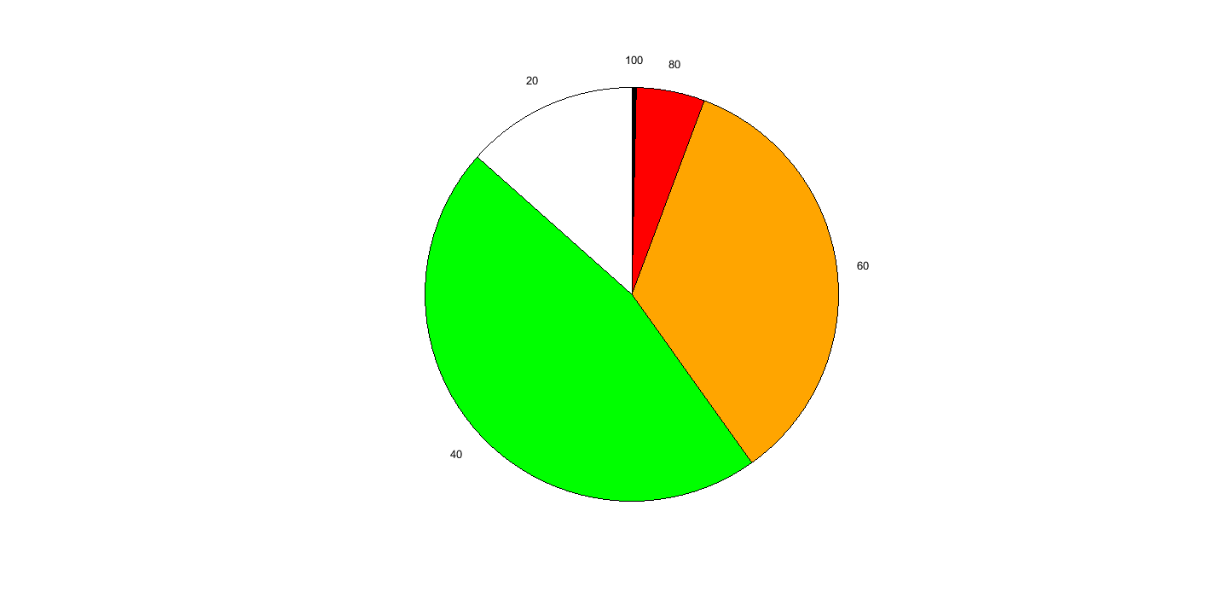

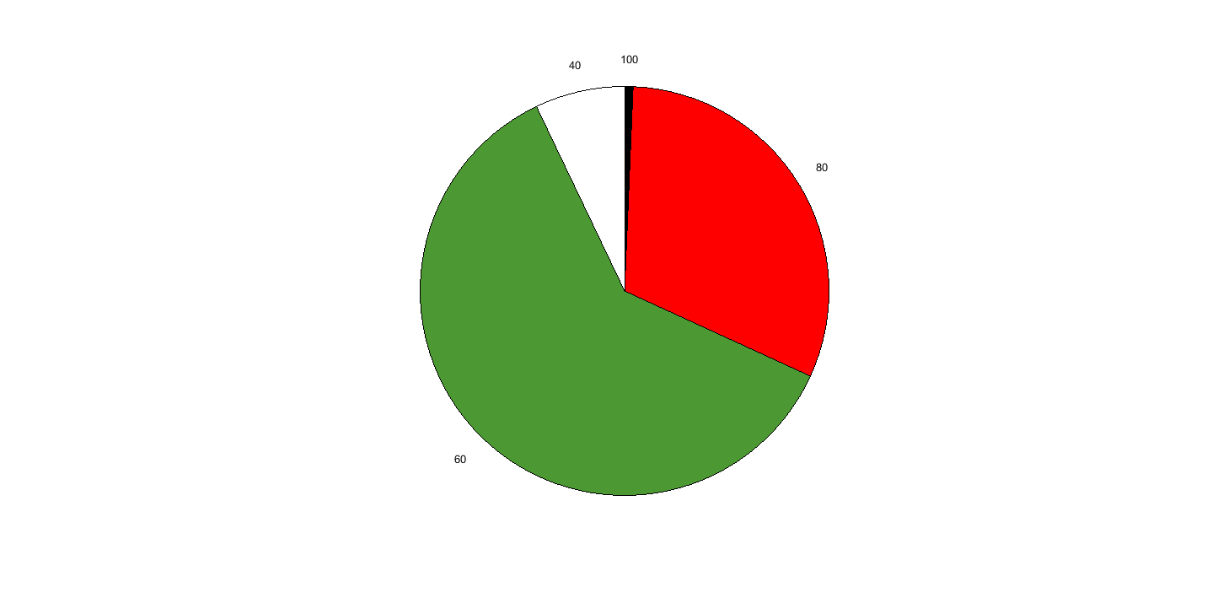

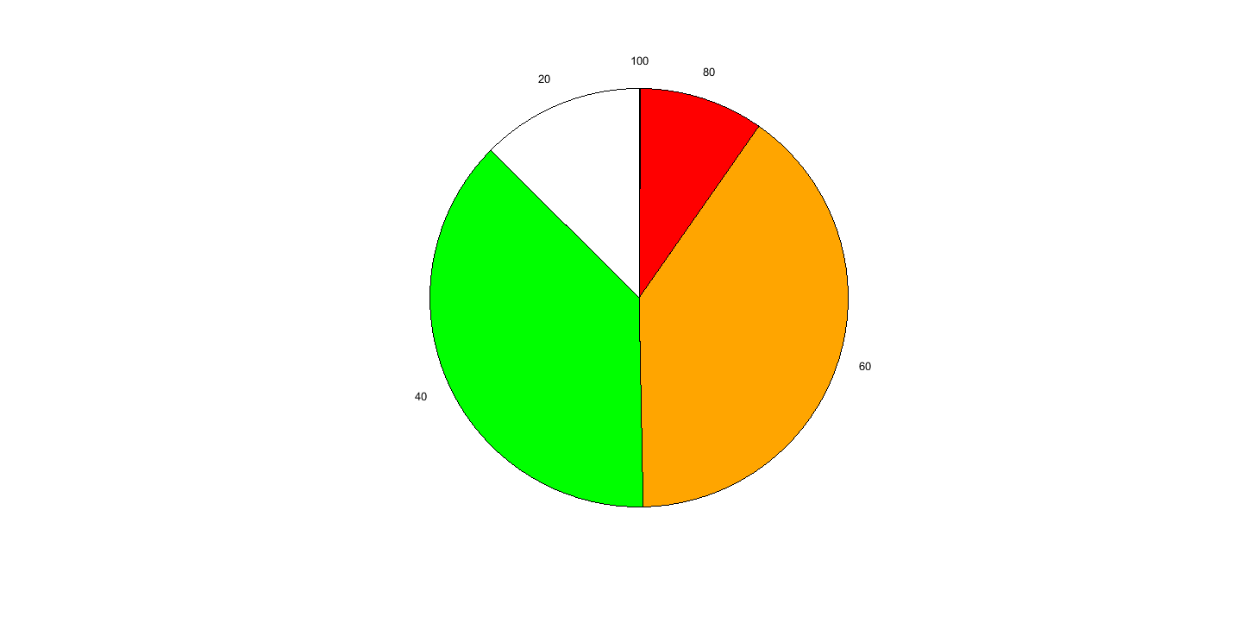

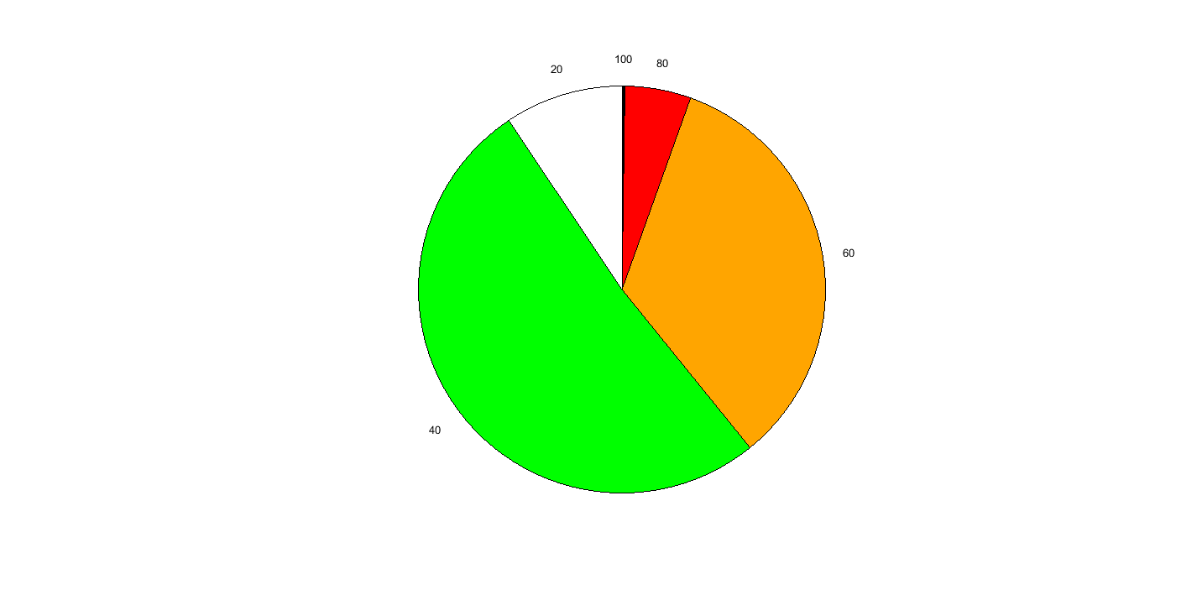

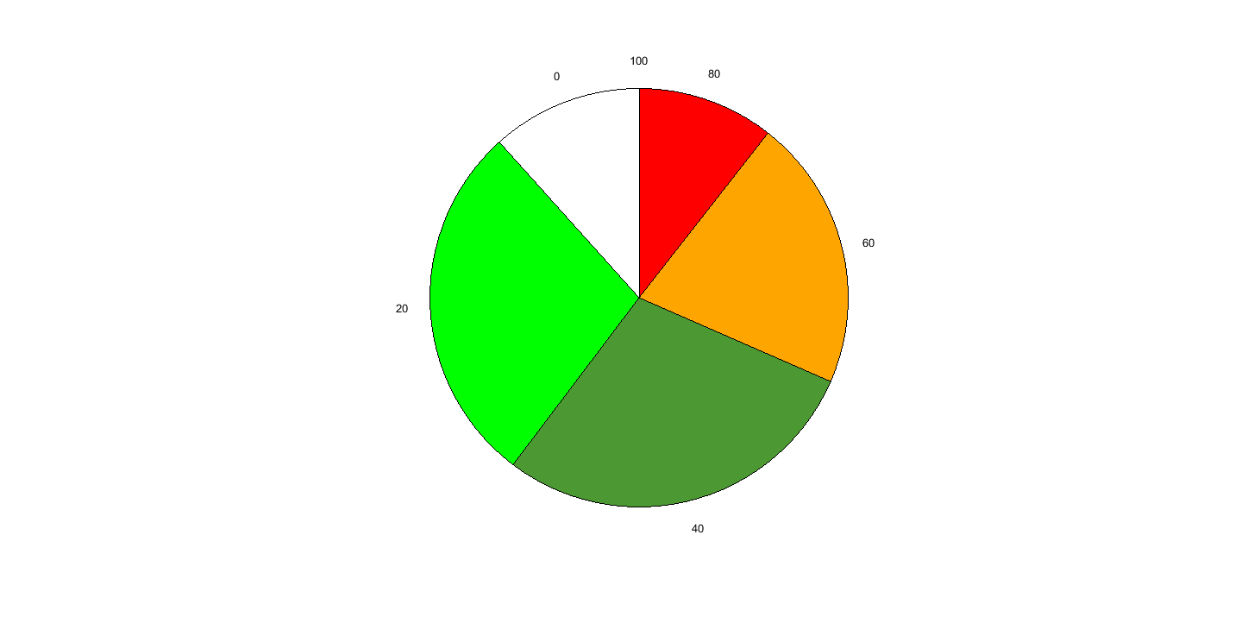

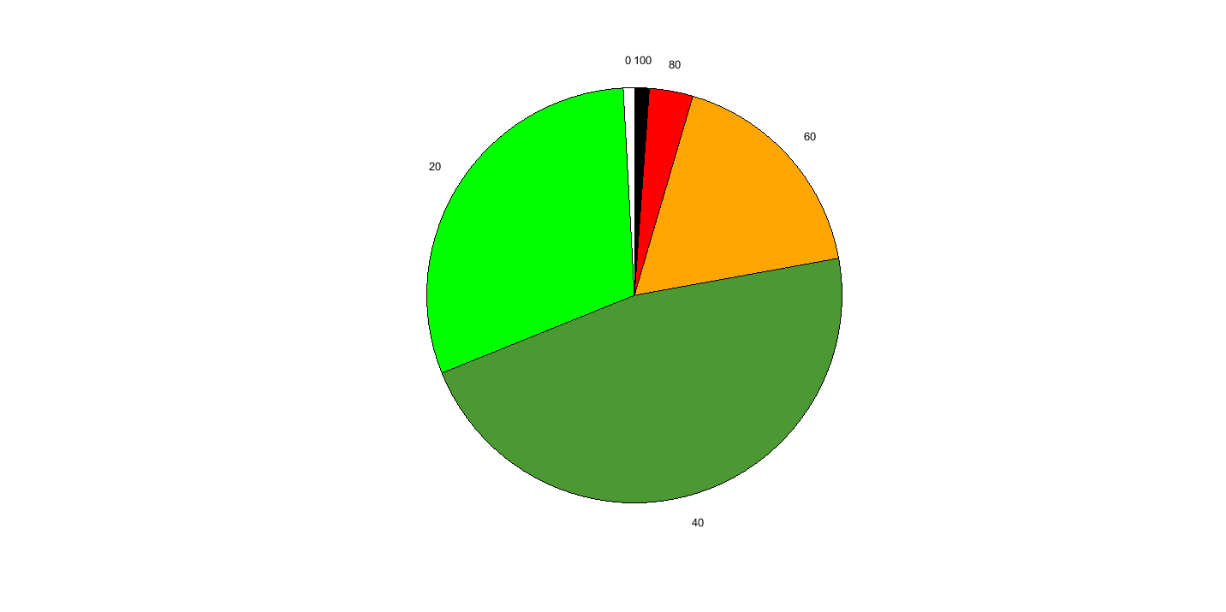

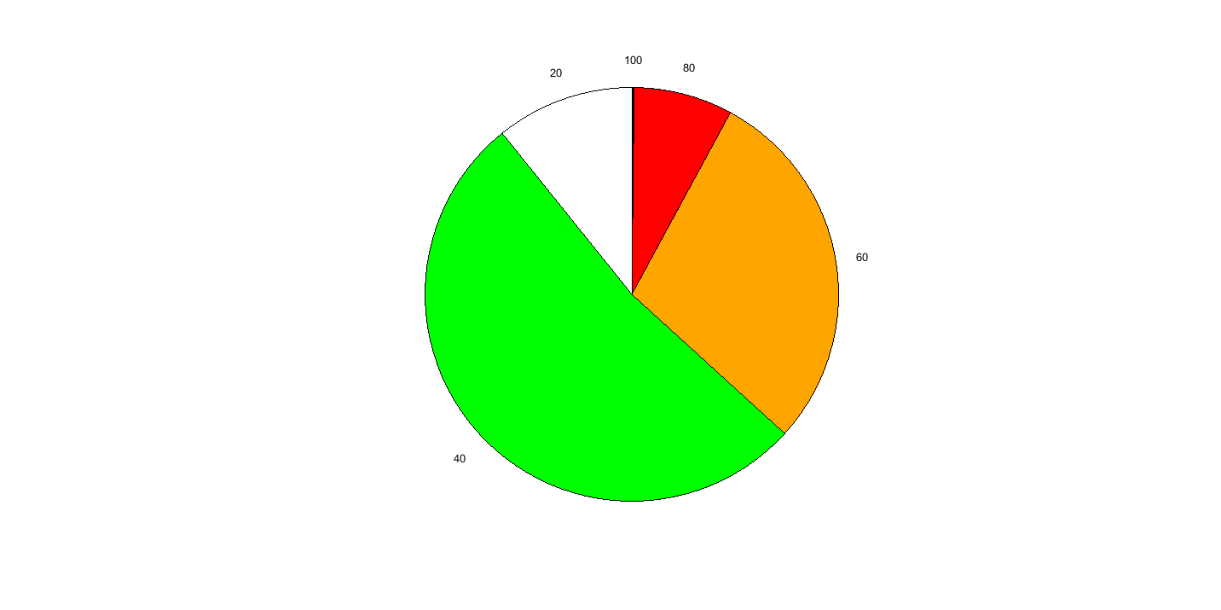

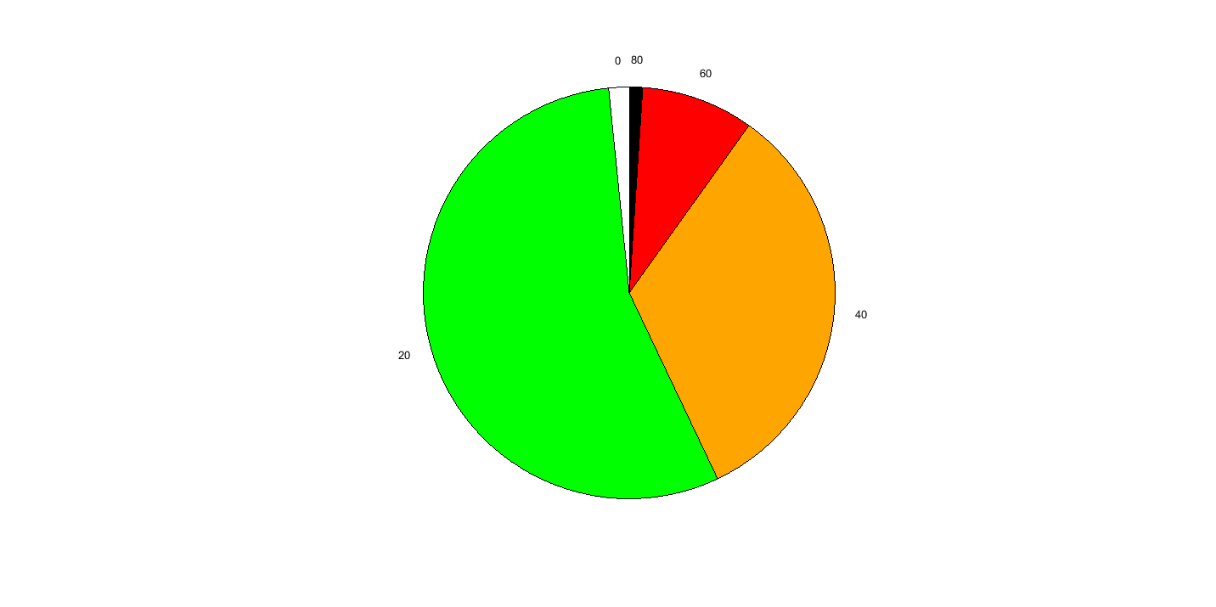

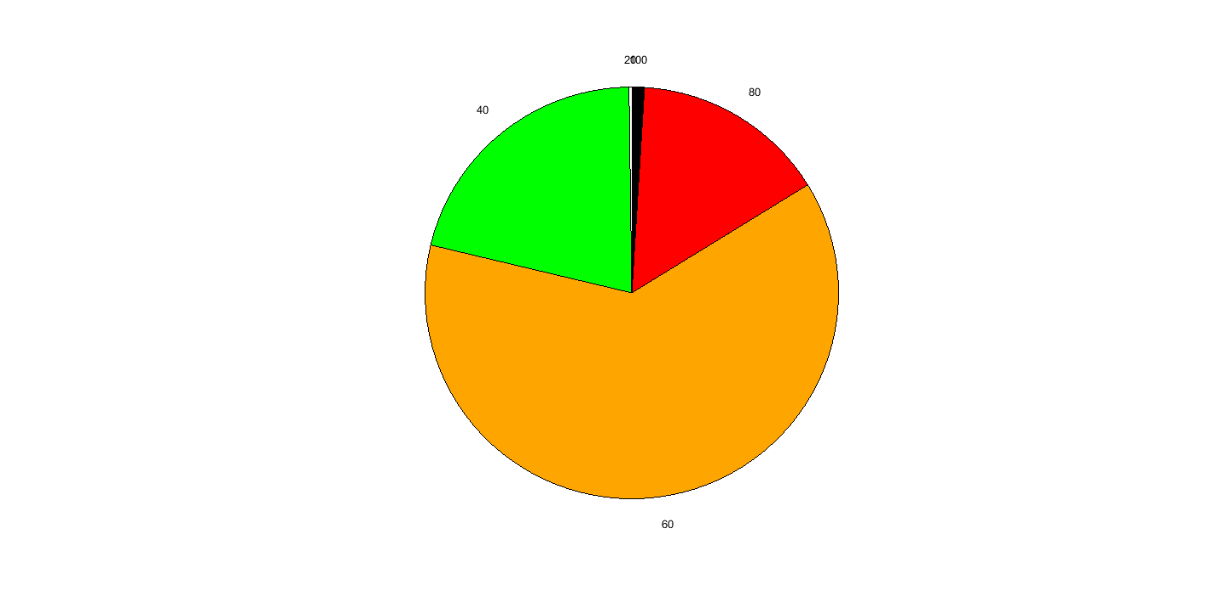

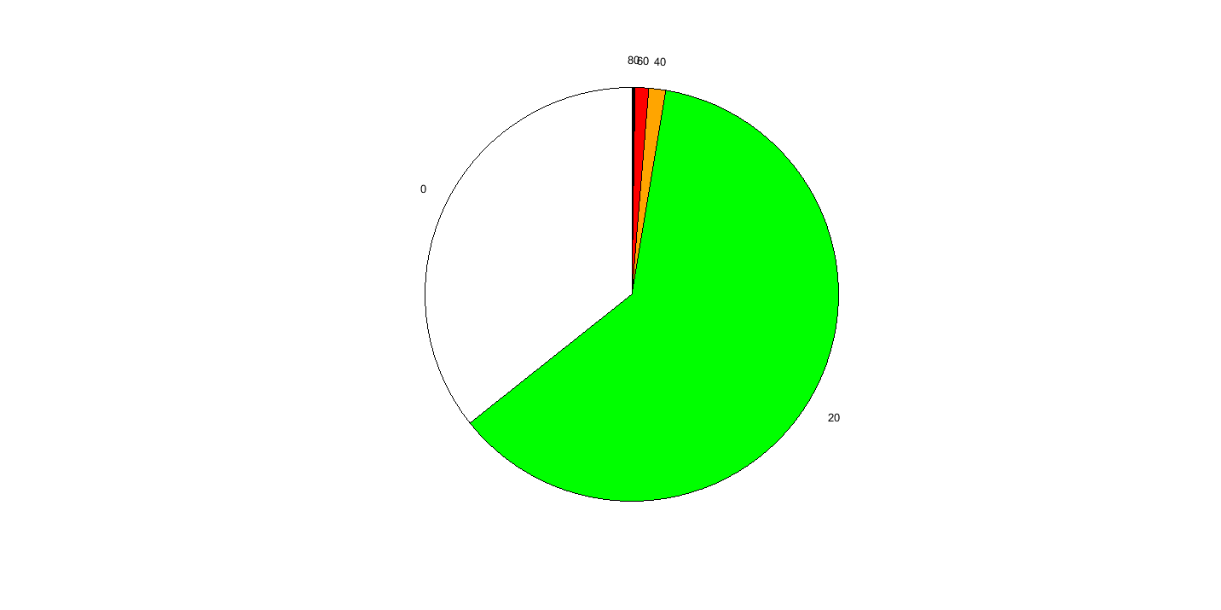

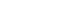

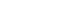

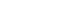

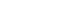

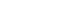

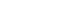

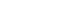

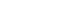

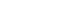

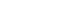

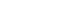


Supplementary video 1. Video fragment of the intervention session held at the athletic facility. The participant will run on an indoor athletic track whilst wearing the biofeedback system. The recording starts prior to the start of the biofeedback condition.

Supplementary audio fragment 1. The discrete levels of noise loudness. A MAX/MSP patch was created to generate the pink noise. The graphical user interface had a slider to categorically increase and decrease the noise level. The noise level evolved from zero (0 %) to maximum (100 %) and again to zero (0 %). The different levels of pink noise were transmitted to the headphone of the participant before commencing the biofeedback run.

Supplementary audio fragment 2. Computer-controlled changes in beats per minute of a self-created song (snippet): 0%, +4%, 0%.

Supplementary audio fragment 3. Transition of a song for music-movement synchronization in tempo. The change illustrates a temporary change of more than 4% in steps per minute, followed by a transition of the song after eight seconds.

Supplementary audio fragment 4. An audio fragment playing at 120 beats per minute with the discrete levels of pink noise sequentially superimposed.

Supplementary datasheet. Metrics of the individual participants. APTA: axial peak tibial acceleration (tibial shock); Δ: difference. Subject names have been removed to preserve confidentiality.
